# A novel XYZ electrospinning that orients the trajectory and collimates the electrified fluid jet of polymer nanofibers by induced electric fields

**DOI:** 10.1371/journal.pone.0308026

**Published:** 2024-08-01

**Authors:** Víctor García-Limón, Oscar E. Aguilar-Mejía, Hector Reyes-Cruz, Ernesto Suaste-Gómez

**Affiliations:** Department of Electrical Engineering, Section Bioelectronics, Center for Research and Advanced Studies (CINVESTAV), Mexico City, Mexico; Minia University, EGYPT

## Abstract

Electrospinning is a process in which high voltage creates nanostructured fibers with random orientation from a polymer solution. A novel electrospinning instrument was designed and constructed, capable of orienting and collimating the trajectory of the electrified fluid jet. The equipment collimates and adjusts the electrified fluid jet in the X-Y directions using deflector plates connected to a variable electric field. Simultaneously, different membrane thicknesses can be selected, i.e., in the Z direction. Additionally, by programming the sinusoidal function generator to perform an X-Y sweep, Lissajous figures (LF) were obtained. SEM images obtained through XYZ electrospinning of PVC and PVDF membranes were used to determine the control achieved over the orientation distribution of the processed nanofibers and the modification of their diameter, with and without applying the electric field to the deflector plates. The nanofibers obtained from the polymeric membranes, which originated after the straight segment of the Taylor cone, did not exhibit a random trajectory and position. Instead, the collimated electrified fluid jet deposited them in a cross pattern (X-Y) on the collector-cathode plate.

## 1 Introduction

The properties of nanometer structures in the form of fibers open up a fascinating panorama under the generic name of nanofibers. Fibers with diameters of less than 100 nanometers are commonly referred to as nanofibers [1]. Electrospinning is a highly versatile route to produce nanofibers [2, 3]. Since then, electrospinning of many synthetic and natural polymers has been developed to obtain uniform and continuous fibers with diameters ranging from a few nanometers to a few micrometers deposited on the collector-cathode plate [4, 5]. During the process, a fluid is passed through a capillary nozzle in an electric field produced by a high voltage source (∼20 kV) [4]; when this reaches a specific value, the surface tension of the tiny droplet formed at the end of the nozzle is overcome, as demonstrated by Taylor [6], exhibiting the Taylor cone associated with an electrified fluid jet. The above gave the guideline for creating polymeric and elongated nanofibers from an electrified fluid jet for application in different modalities [7]. This work aimed to create electrospinning equipment capable of orienting and collimating to a controlled position by inducing an electric field acting on the fluid jet deposited on the collector-cathode plate. The orientation or deflection of the fiber-electrified fluid jet was performed by an electrostatic deflection system after the fiber or positively charged polymer jet [8] leaves the tip of the Taylor cone, formed by two electrodes in the form of fixed deflector plates in X and Y direction, placed closely, perpendicularly to the center of the fiber electrified fluid jet. These deflection plates are connected to a signal function generator to be polarized, and they are used to generate different types of electrical waveforms over a wide range of frequencies. Some of the most common waveforms the function generator produces are the sine wave, square wave, triangle wave, and sawtooth shapes. Thus, with negative electric waveforms or a variable negative electric field, the electrified positive fluid jet is oriented in the chosen X-Y position, obtaining collimated electrospun membranes with different geometrical arrangements or shapes. Furthermore, it was possible to obtain results like those obtained in the LF. Simultaneously, selecting different membrane thicknesses, i.e., in the Z direction, is possible; this led to the development of an XYZ electrospinning equipment by orienting the current path of the electrified fluid jet of fibers diameter, with and without applying the electric field to the deflector plates. The nanofibers obtained from the polymeric membranes, which originated after the straight segment of the Taylor cone, did not exhibit a random trajectory and position. Instead, the collimated electrified fluid jet deposited them in a cross pattern (X-Y) on the collector-cathode plate.

## 2 Sample preparation and experimental setup

### 2.1 Sample preparation

The nanofiber membranes were created using polyvinylidene fluoride (PVDF), a synthetic resin polymerizing vinylidene fluoride (CH2 = CF2). PVDF is a rigid plastic resistant to flame, electricity, and most chemicals. On the other hand, PVDF is a polymeric smart material with interesting piezoelectric, pyroelectric, and ferroelectric properties [9]. PVDF membranes are flexible, lightweight, biocompatible, and chemically stable [9, 10]. Furthermore, PVDF responds to pressure, electric signals, temperature, sound, light, and moisture [11]. Due to PVDF’s biocompatibility and humidity sensitivity, PVDF is an excellent candidate as a sensor for biomedical applications. The development of prosthesis fabricated in polyvinylidene fluoride by a 3D printer [12]. The PVDF/DMF (Dimethylformamide) solution was prepared as follows: a 20 wt.% composition was achieved by dissolving PVDF powder in DMF. The mixture was then kept at 60°C for 1 hour to ensure homogeneity. PVDF powder and DMF were purchased from Sigma Aldrich (Saint Louis, MO, USA). The other material used, Polyvinyl chloride (alternatively: poly(vinyl chloride) abbreviated: PVC [13] to manufacture the PVC membranes was obtained employing an adhesive-based solution of PVC resins in organic solvents, Ref.: 501027, Ceys Co. from which a sample was obtained and placed in the XYZ electrospinning system to get the membrane.

### 2.2 Experimental setup

**Fig 1:**
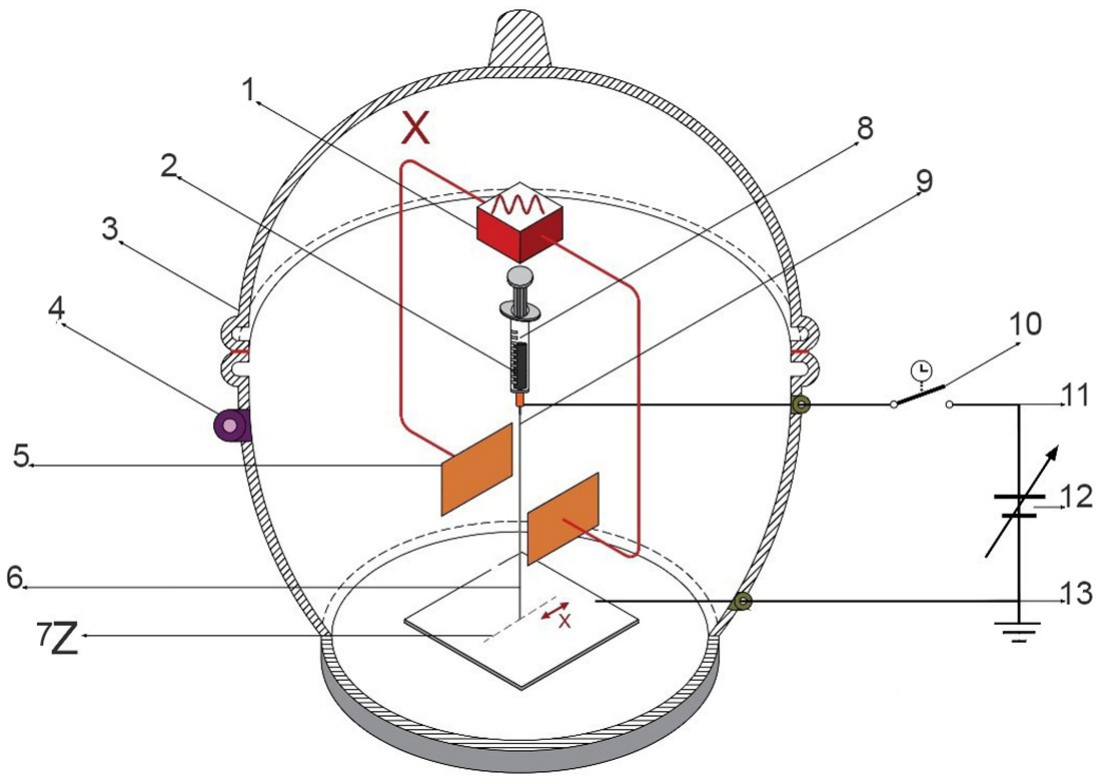
Assembly of XZ electrospinning system. 1) Function generator, 2) Polymer, 3,4) Electrical isolation, 5) Deflector plates X, 6) Electrified fluid jet trajectory,7) Thickness Z, 8) Polymer injector, 9) Taylor cone (straight segment), 10) Timer switch, 11) Anode, 12) High voltage 2 to 20 kV, 13) Collector-cathode plate.

**Fig 2:**
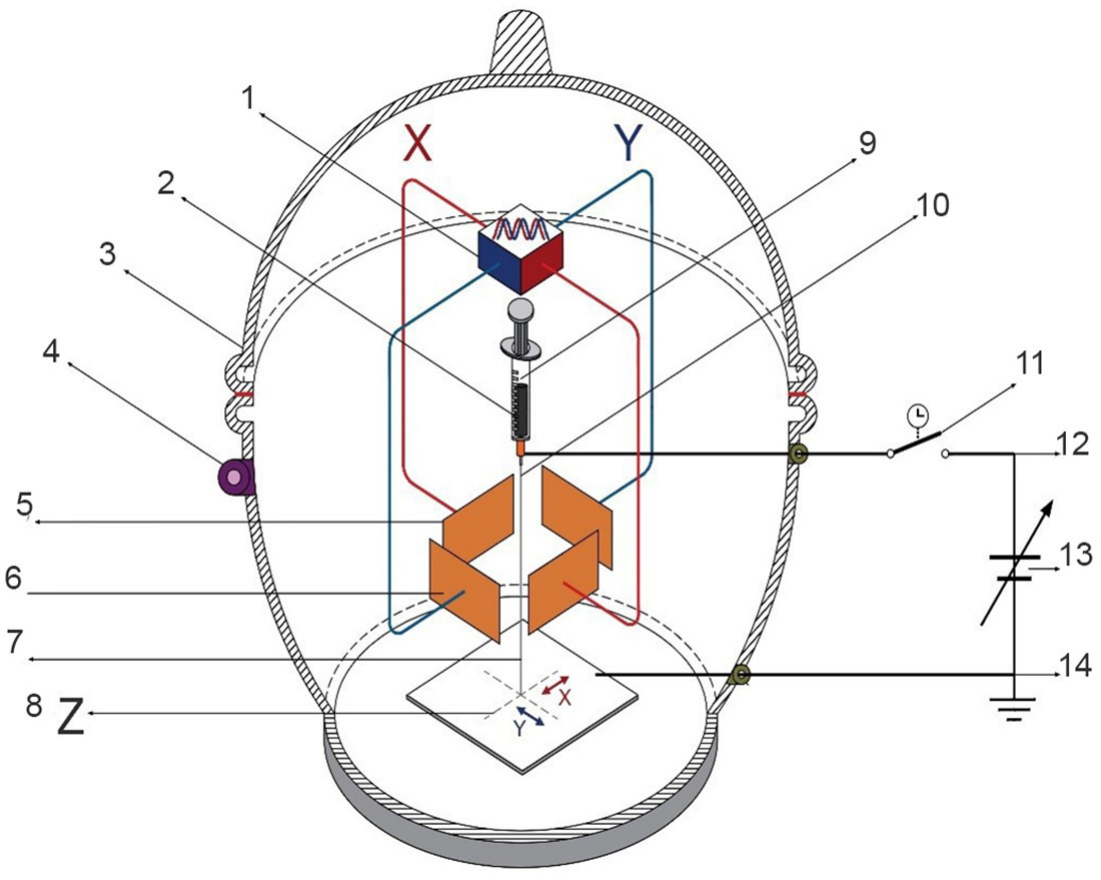
Assembly of XYZ electrospinning system. 1) Function generator, 2) Polymer, 3,4) Electrical isolation, 5) Horizontal deflector plates X, 6) Vertical deflector plates Y, 7) Electrified fluid jet trajectory, 8) Thickness Z, 9) Polymer injector, 10) Taylor cone (straight segment), 11) Timer switch, 12) Anode, 13) High voltage 2 to 20 kV, 14) Collector-cathode plate.

**Fig 3:**
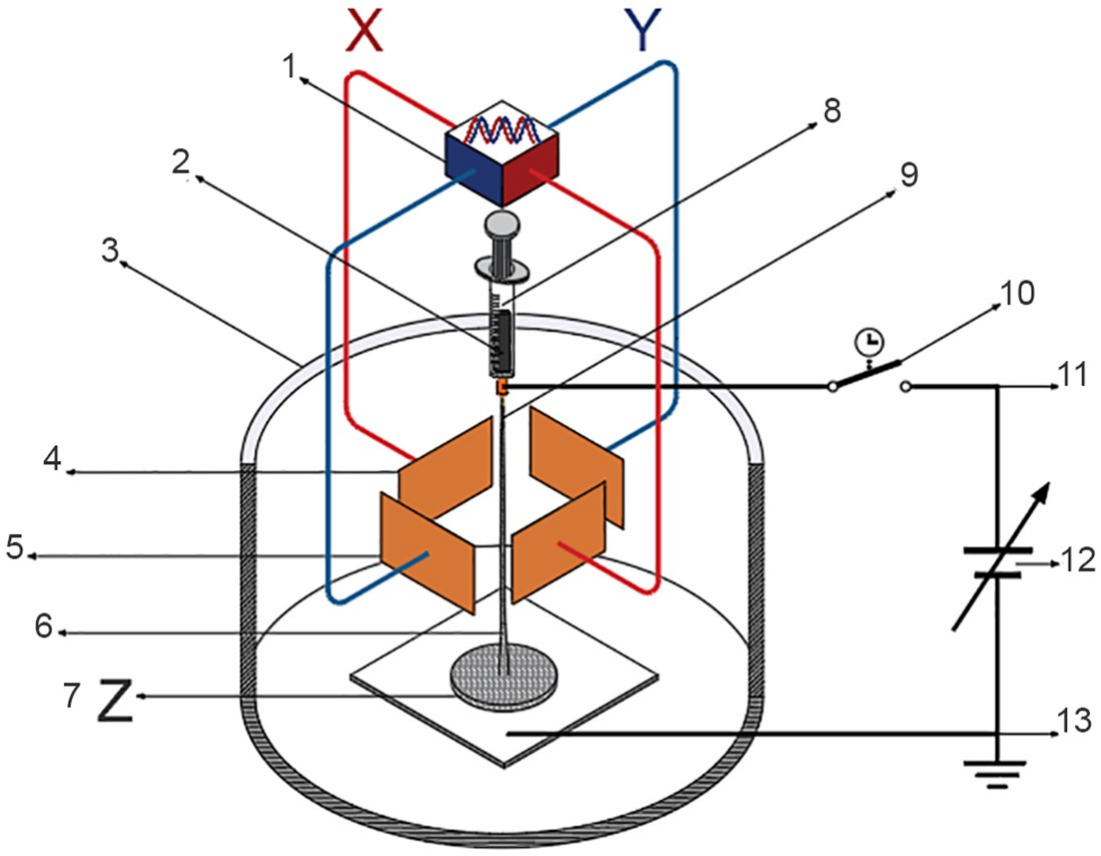
The assembly of the LF-XYZ electrospinning system follows the LF trend. 1) Function generator X(t) = A sin(*ω*t), Y(t) = B sin(Ωt + *δ*), the deflector plates for X and Y are biased with negative sinusoidal signals of -10 Vpp at a frequency of 1 Hz, phase-shifted by 90°, 2) Polymer or substance, 3) Electrical isolation, 4) Horizontal deflector plates X, 5) Vertical deflector plates Y, 6) Positively electrified fluid jet trajectory, 7) Thickness Z of LF, 8) Polymer injector, 9) Taylor cone (straight segment), 10) Timer switch, 11) Anode, 12) High voltage 2 to 25 kV, 13) Collector-cathode plate.

The XYZ electrospinning developed for obtaining polymer membranes or “prints” made of ultra-thin branched fibers elaborated using the mentioned technique of modulated and collimated electrospinning, i.e., avoiding randomness in the trajectory of the electrified fluid jet in the polymeric membrane mesh. The way to carry out these non-random membranes, as far as their direction and position, was experimentally tested. Thus, the trajectory of the positively electrified fluid jet of the polymer nanofiber was modulated and collimated using an electrostatic deflection system induced with a negative sinusoidal signal in the positively charged jet of the polymer as it leaves the tip of the Taylor cone. This electrostatic modulation system consists of two pairs of copper plates or electrodes with a thickness of 1 mm of 72 x 38 mm, separated at a distance between 45 and 30 mm as deflectors placed perpendicularly for the X and Y direction, which in turn, were polarized with an electric field employing a sinusoidal signal generator with peak amplitude from 5 to 20 V, shifting the signal or offset to the negative plane, at a frequency from 1 to 10 Hz. The distance from the nozzle that initiates the Taylor cone to the collector-cathode plate of the high-voltage source was 110 mm. Polymeric membranes featuring nanofibers were produced through the application of two sinusoidal signals with varying frequencies, phases, and amplitudes, induced on the X-Y deflector plates, as illustrated in the diagrams of Figs 1 and 2. Also, similar to the LF, polymeric membranes were produced, Fig 3. The thickness or overlap of the membrane layers, modulated by the exposure time, gives the trajectory Z. A high voltage of 18 kV was used for XYZ electrospinning. Fig 1 shows the electrospinning setup for generating the jet of collimated and directed electrified fibers in the X position employing horizontal deflection plates X and the thickness Z of the membrane.

Fig 2 shows the electrospinning setup for generating the jet of collimated and directed electrified fibers in the XY position, employing horizontal deflection plates X, vertical deflection plates Y, and the thickness Z of the membrane.

Fig 3 illustrates the experimental arrangement for obtaining polymer or substance membranes, following the trend of the LF, where the X and Y deflector plates were polarized with two negative sinusoidal signals of -10 Vpp at a frequency of 1 Hz, phase-shifted by 90°, out of phase.

In this context, the differences, advantages, and disadvantages of the XYZ electrospinning system are summarized in Figs 1–3:

Fig 1: XZ electrospinning, configuration Fig 1 illustrates the electrospinning setup that generates a collimated and directed jet of electrified fibers in the X position, employing horizontal X deflector plates. The excitation voltage applied to the deflector plates is -10 Vpp at a frequency ranging from 1 to 10 Hz, with Z denoting the membrane thickness.

Fig 2: XYZ electrospinning, configuration Fig 2 demonstrates the electrospinning setup for producing a collimated and directed jet of electrified fibers in the XY position, utilizing horizontal X deflector plates and vertical Y deflector plates. The excitation voltage applied to the X-Y deflector plates is -10 Vpp at a frequency ranging from 1 to 10 Hz, with Z representing the membrane thickness.

Fig 3: LF-XYZ electrospinning configuration Fig 3 shows the experimental setup for membrane fabrication following the LF. Here, the deflector plates for X and Y are biased with negative sinusoidal signals of -10 Vpp at a frequency of 1 Hz, phase-shifted by 90°. Z is indicative of the membrane thickness. LF have been an important tool in physics and engineering; they allow for the graphical analysis and interpretation of the interaction of two perpendicular motions on a particle [14]. Additionally, it is possible to generate LF made of nanofibers by manipulating the X and Y signals, i.e., in terms of two waves with different possible amplitudes and frequencies, we can write the following for the X and Y coordinates used to display the LF:

X(t)=Asin(ωt),Y(t)=Bsin(Ωt+δa)

Consequently, as the phase of the two waves (of the same frequency) varies, the resulting output shape transforms. The output shape is a circle when the waves are at a phase angle of *π*/2. The output shape is a straight line when the waves are in phase. When the waves are at a phase angle of exactly *π*/2, the output shape is a distorted ellipse. Various distorted ellipses can be observed between these two extremes [15].

## 3 Results

### 3.1 Conventional electrospinning setup

**Fig 4:**
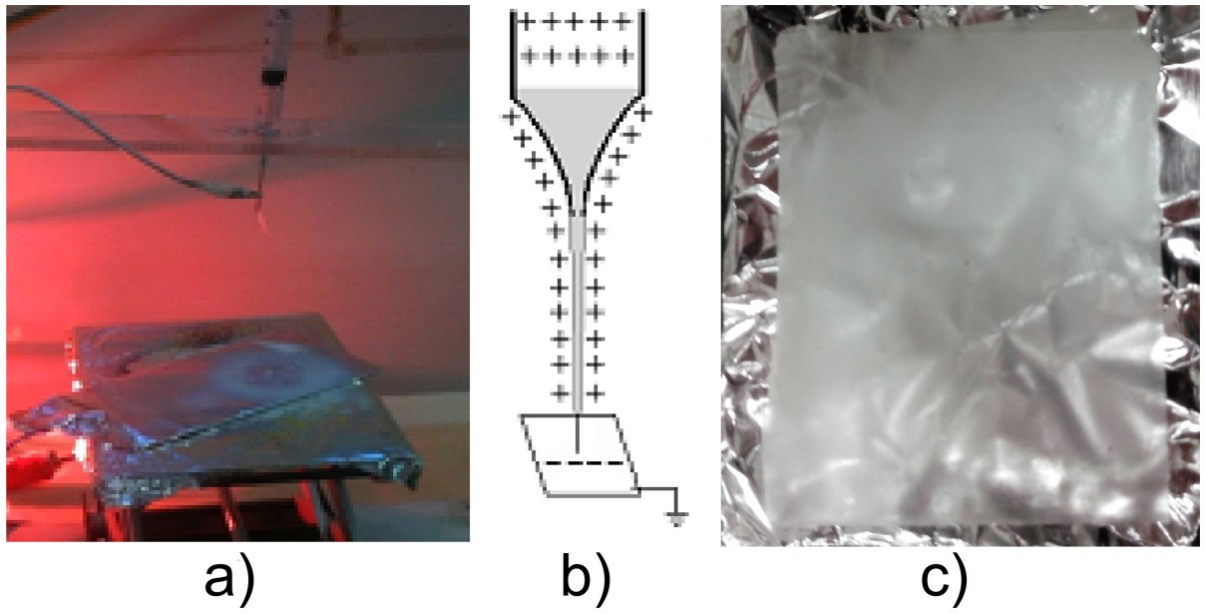
a) Experimental arrangement of electrospinning of polymeric nanofiber membranes in a random, unmodulated fashion of PVDF membrane. The positive jet polarity of the polymer jet (Taylor cone) used PVDF is exhibited by b) and c) PVDF randomly shaped polymeric nanofiber membrane on an aluminum collector-cathode plate.

The results show the experimental arrangement of conventional electrospinning, randomly without modular Fig 4, where a PVDF nanofiber membrane was obtained. The distance between the polymer injector and the collector-cathode plate was 110 mm, the positive polarity of the polymer jet (Taylor cone) used PVDF or PVC is shown, and the membrane deposited on the collector-cathode plate with a thickness as shown in Fig 11 (Z vs Time) (c). The high voltage was 18 KV from electrospinning.

### 3.2 XZ electrospinning setup

**Fig 5:**
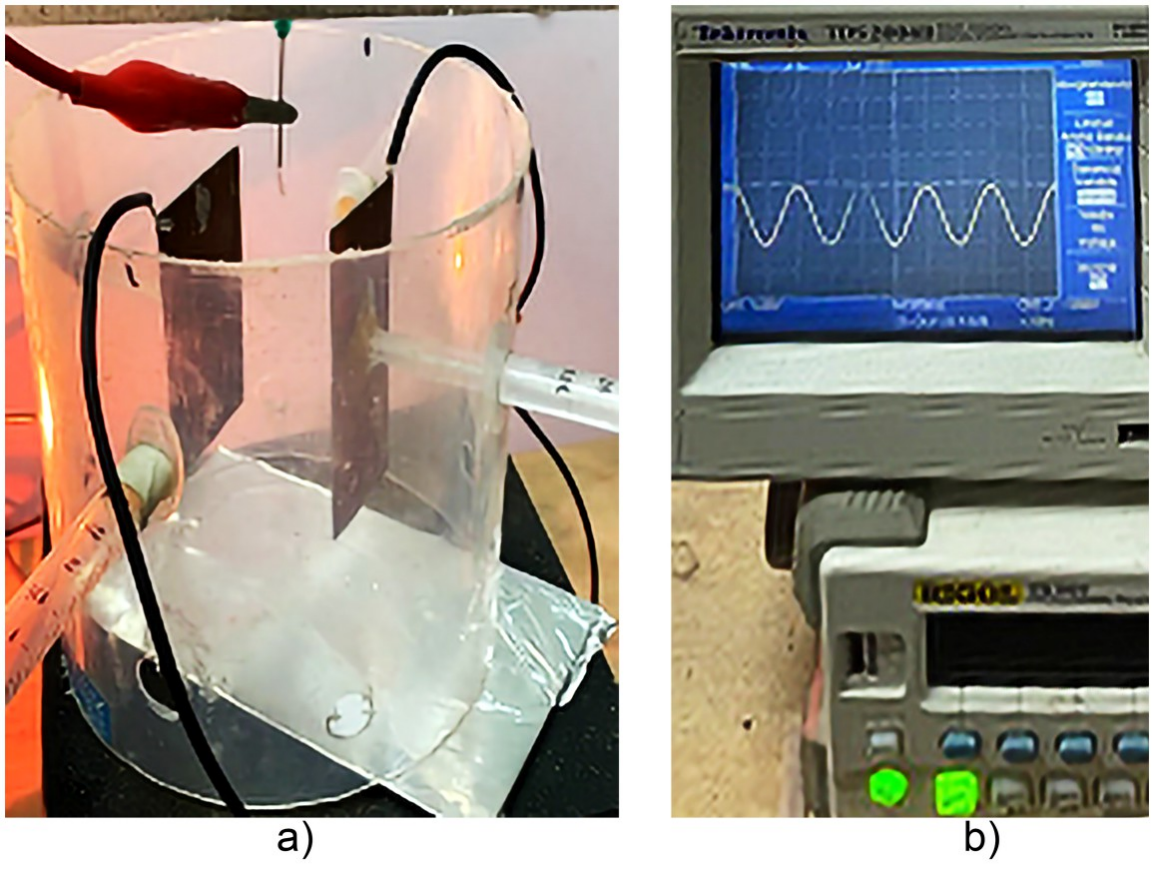
Shows the experimental arrangement based on XZ electrospinning, a) two electrodes or deflector plates on direction X separated with 110 mm where it was excited b) with a sinusoidal signal of -10 Vpp, at a frequency of 1 Hz. The high voltage was 18 KV from the electrospinning for 4 minutes.

Fig 5 shows the experimental arrangement; the polymer for the test was PVC resin, illustrated in the schematic in Fig 1, a) two electrodes or deflector plates separated with 110 mm where it was excited b) with a sinusoidal signal with peak-to-peak amplitude of 10 V shifting the signal or offset to the negative plane (-10 Vpp) at a frequency of 1 Hz. The high voltage was 18 KV from the electrospinning for 4 minutes, with a thickness Z, as shown in Fig 11.

**Fig 6:**
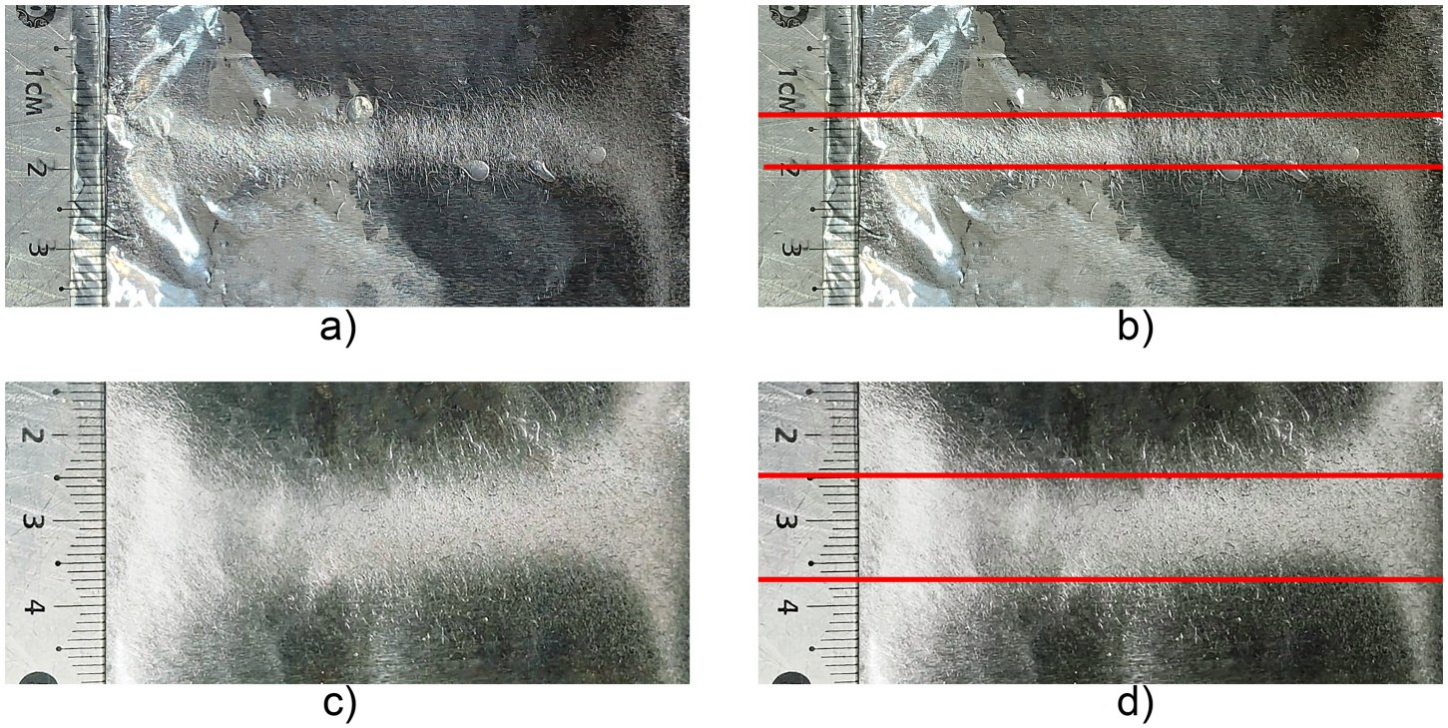
Illustrates the deposits of the polymeric membranes in the X direction based on Fig 1, with different dimensions in terms of width deposited of 15 mm (a,b) y 6 mm (c,d) with a distance between deflector plates of 45 mm y 30 mm respectively, with a sinusoidal signal of -10 Vpp, at a frequency of 1 Hz. The thickness (Z) of the membranes was 248 *μ*m (PVC, Fig 11).

**Fig 7:**
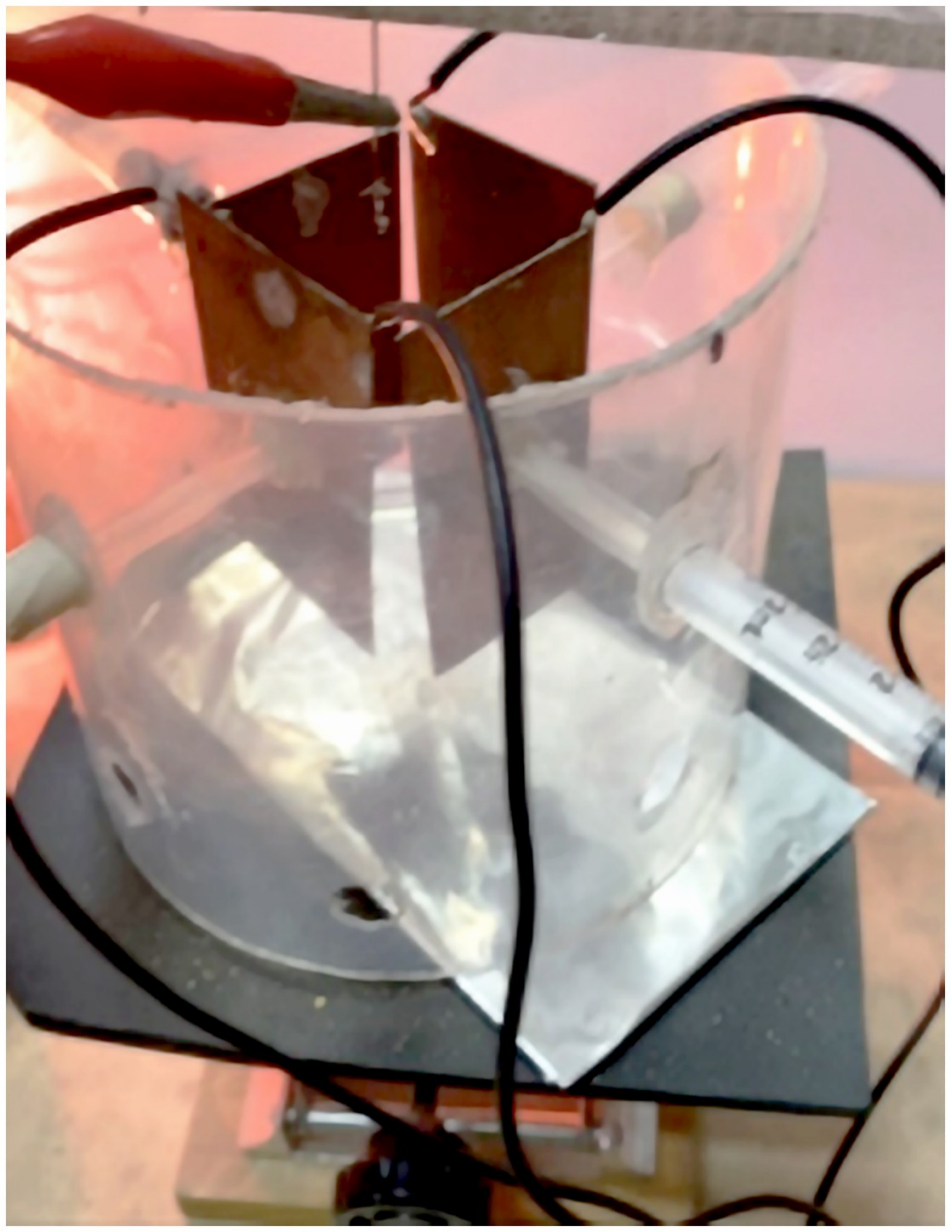
Shows the experimental setup that was used based on Fig 2, consisting of a pair of deflector plates for the X and Y directions, as well as the results of the electrospinning of the polymer in the X-Y paths on the aluminum cross-shaped collector-cathode plate.

**Fig 8:**
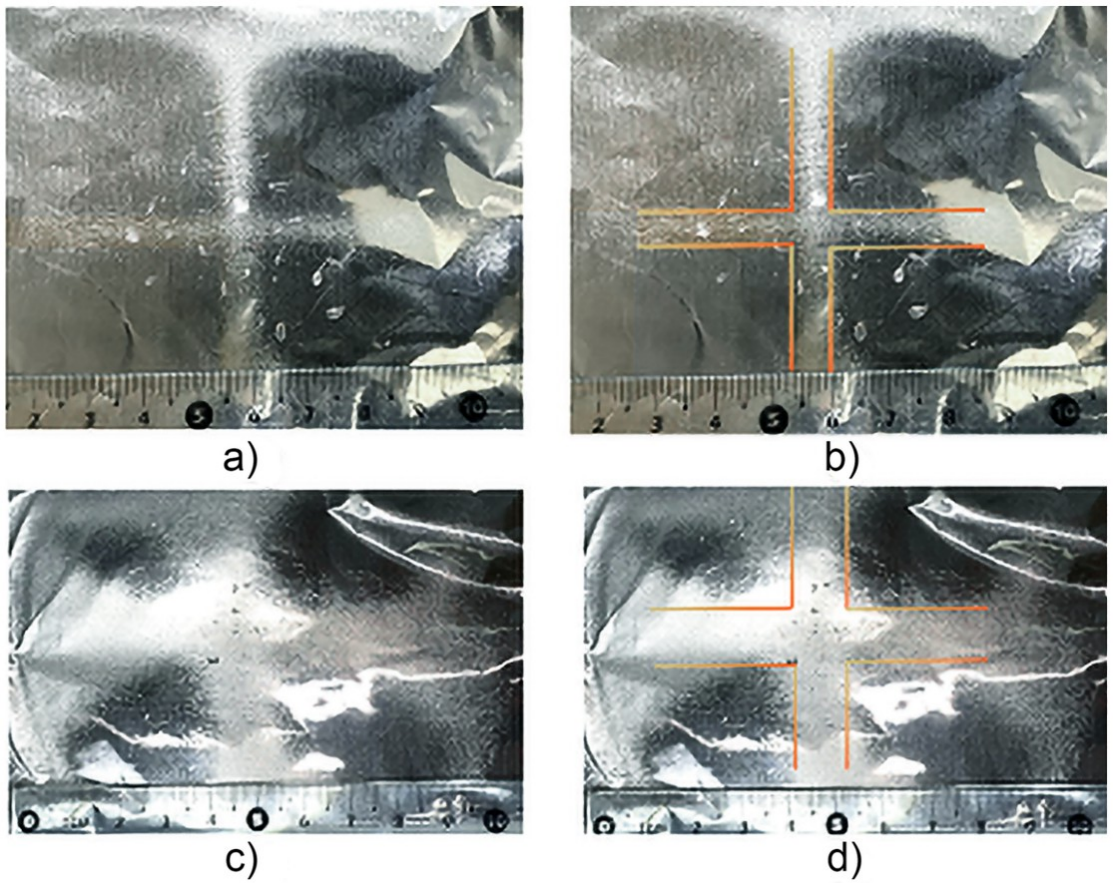
Illustrates the cross-shaped polymeric membranes deposition with widths of 7 mm (a,b) and 12 mm (c,d) with a distance between deflector plates of 30 mm and 45 mm, respectively, both in X and Y. The Z thickness was 248 *μ*m (PVC, Fig 11).

Fig 6 illustrates the deposition of the polymeric membranes in the XZ direction based on Figs 1 and 5, with different dimensions in terms of deposited width of 15 mm (a,b) and 6 mm (c,d) with a distance between deflector plates of 45 mm and 30 mm respectively, with -10 Vpp, at a frequency of 1 Hz. On the aluminum collector-cathode plate, the Z thickness of the polymeric membranes was 248 μm (Fig 11). Fig 7 shows the experimental setup used, consisting of a pair of deflector plates for the X and Y directions, as well as the results of the electrospinning of the polymer in the X-Y paths on the aluminum cross-shaped collector-cathode plate, which was obtained using a sinusoidal signal of -10 Vpp, at a frequency of 1 Hz. Also shown are the cross-shaped membranes deposition on the aluminum collector-cathode plate with different membrane widths as illustrated in Fig 8, (a,b) 7 mm and (c,d) 12 mm with a distance between deflector plates of 30 mm and 45 mm respectively, both in X and Y. The Z thickness was 248 *μ*m (PVC, Fig 11).

### 3.3 XYZ electrospinning setup to follow LF trajectories

**Fig 9:**
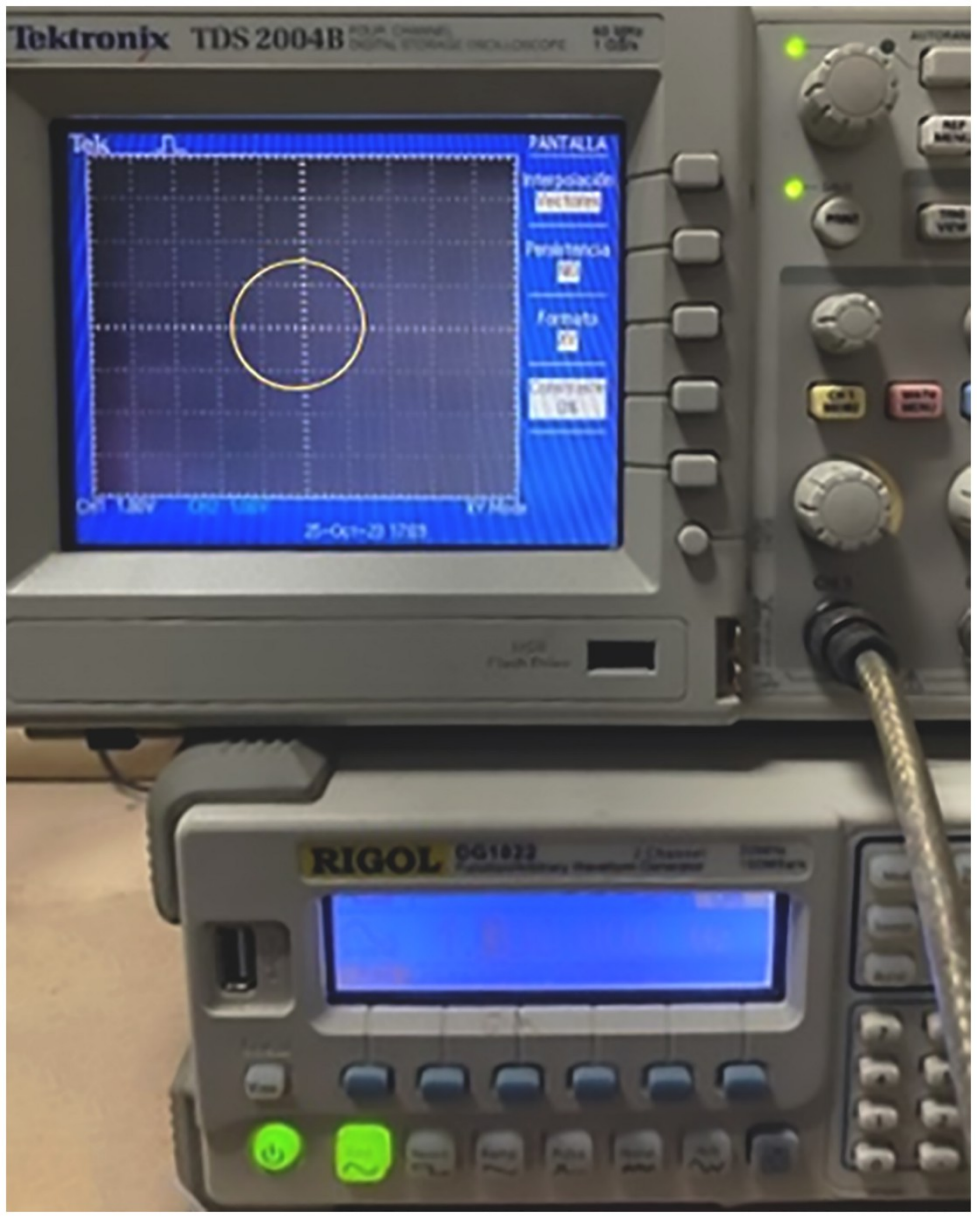
Describes the instrumentation used to obtain a circular-shaped LF. The phase difference between the two sinusoidal waves with amplitudes of -10 Vpp signals at 1 Hz for X and Y was 90°.

**Fig 10:**
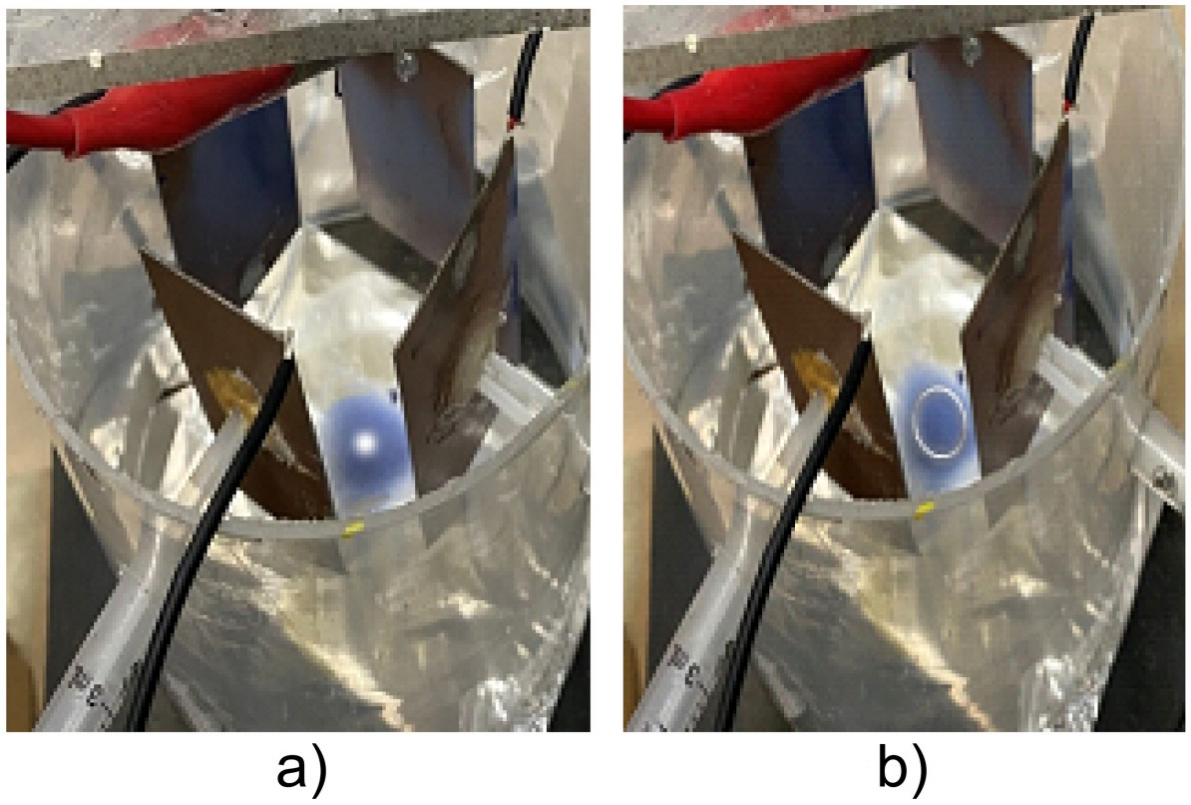
Illustrates the deposition or print of the PVC-pigmented resin electrospun membrane on the collector-cathode plate based on Fig 3, (a) clearly shows the circular trend of the positively charged polymer jet width trajectory in the electrospun membrane print (b), similar to LF.

Continuing in this context, considering the concept for obtaining the LF that have been a very important tool in physics and engineering, these allow graphically analyzing and interpreting the interaction on a particle or electron beam of two perpendicular oscillatory motions of different frequencies [15]. Furthermore, emulating the obtaining of the “printed” or deposited LF on the collector-cathode plate, carried out by induction of a negatively charged electric field acting on the X and Y plates, which in turn impinge on the positively charged polymer jet exiting the Taylor cone (Fig 4b), when using the proposed XYZ electrospinning, illustrated in the schematic in Fig 3. Also, experimentally, it was tested, where the X and Y deflector plates were polarized with two sinusoidal waves, X(t) = A sin(*ω*t) and Y(t) = B sin(*Ω*t + *δ*), with amplitude of -10 Vpp to A and B, at a frequency of 1 Hz out of phase 90°. Likewise, Fig 9 describes the instrumentation that was used to obtain a circular biased LF. The results obtained using XYZ electrospinning were satisfactory, as shown in Fig 10.

The deposition of the PVC-pigmented resin electrospun membrane on the cathode-collector plate is illustrated in Fig 10, a) clearly showing the circular trend of the positively charged polymer jet width trajectory in the electrospun membrane print b), similar to LF.

### 3.4 Thickness Z

**Fig 11:**
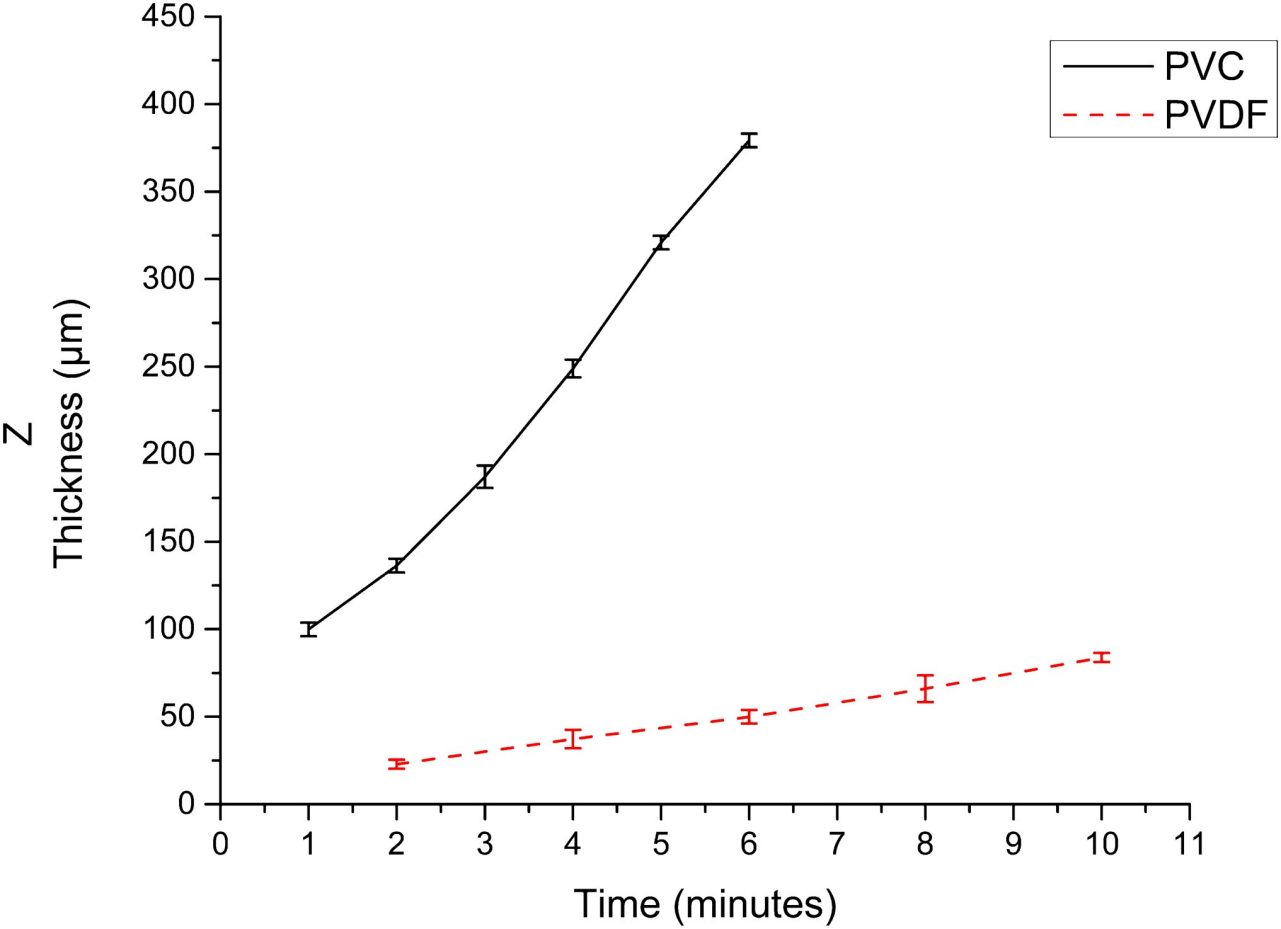
Exposure time against the corresponding Z, PVC-based resin, and PVDF polymer.

Concerning the thicknesses (Z) obtained from membranes of a polymer based on PVC resins and PVDF. The graph Fig 11 shows the electrospinning exposure time in minutes vs. the corresponding Z thickness in μm is illustrated for PVC resin and PVDF.

### 3.5 Results of the effect of the additional electrical field on the nanofibers diameter and the orientation of the produced nanofibers by XYZ electrospinning on PVC and PVDF membranes

**Fig 12:**
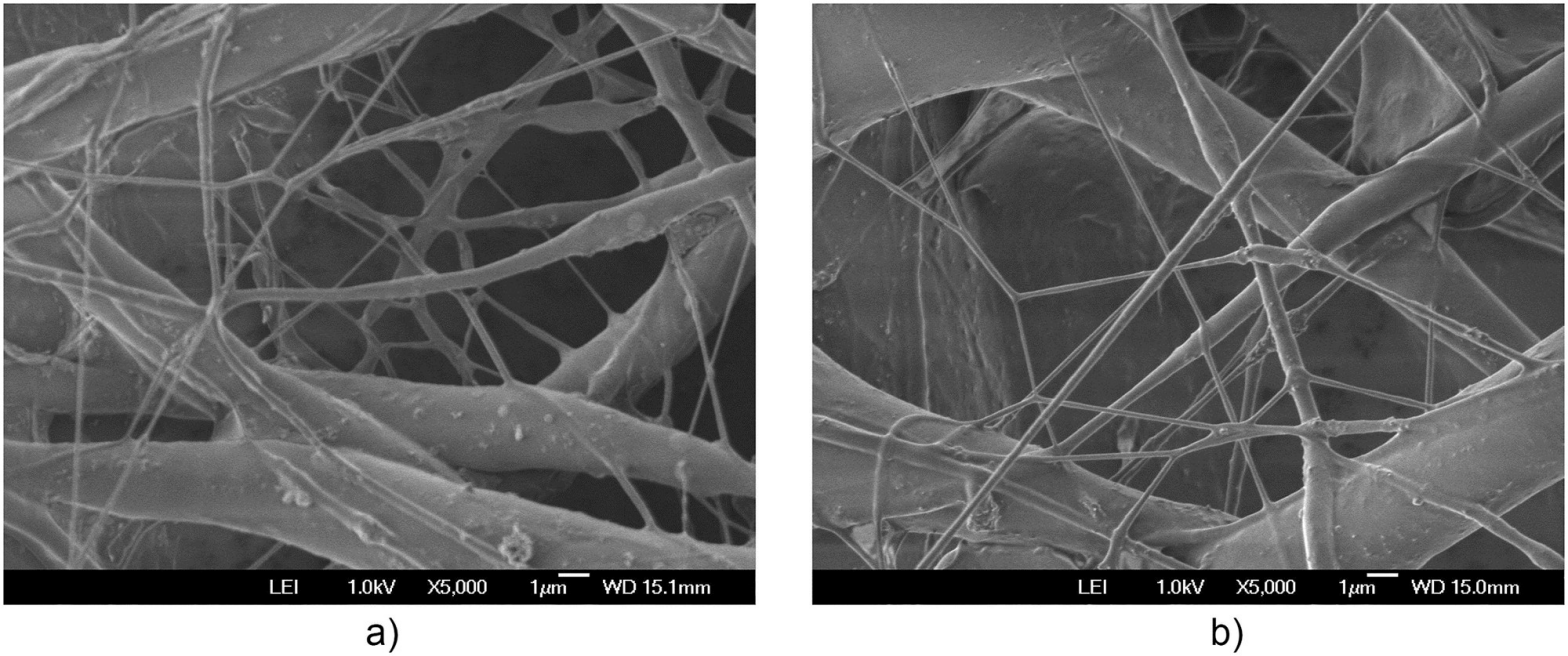
PVC SEM x5000, a) central zone with electric field b) end zone without applying the electric field.

**Fig 13:**
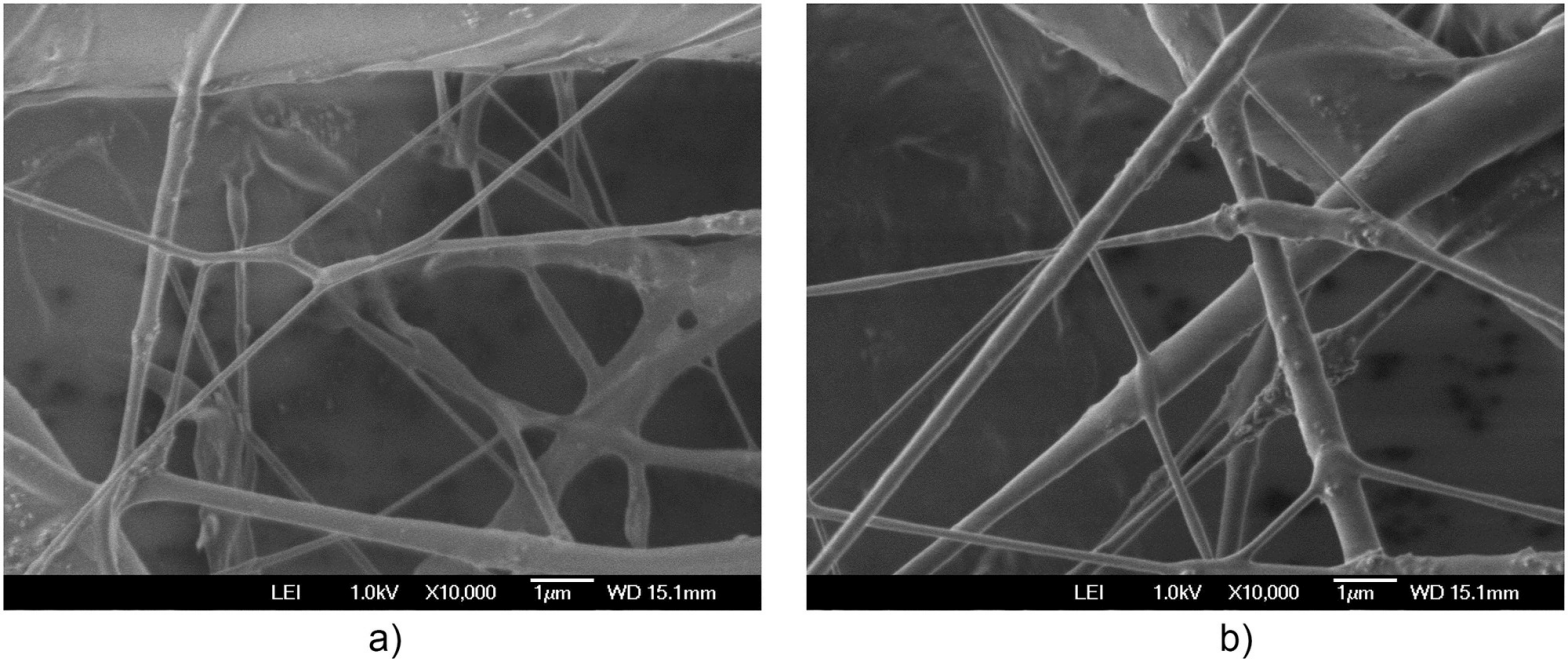
PVC SEM x10000, a) central zone with electric field b) end zone without applying the electric field.

**Fig 14:**
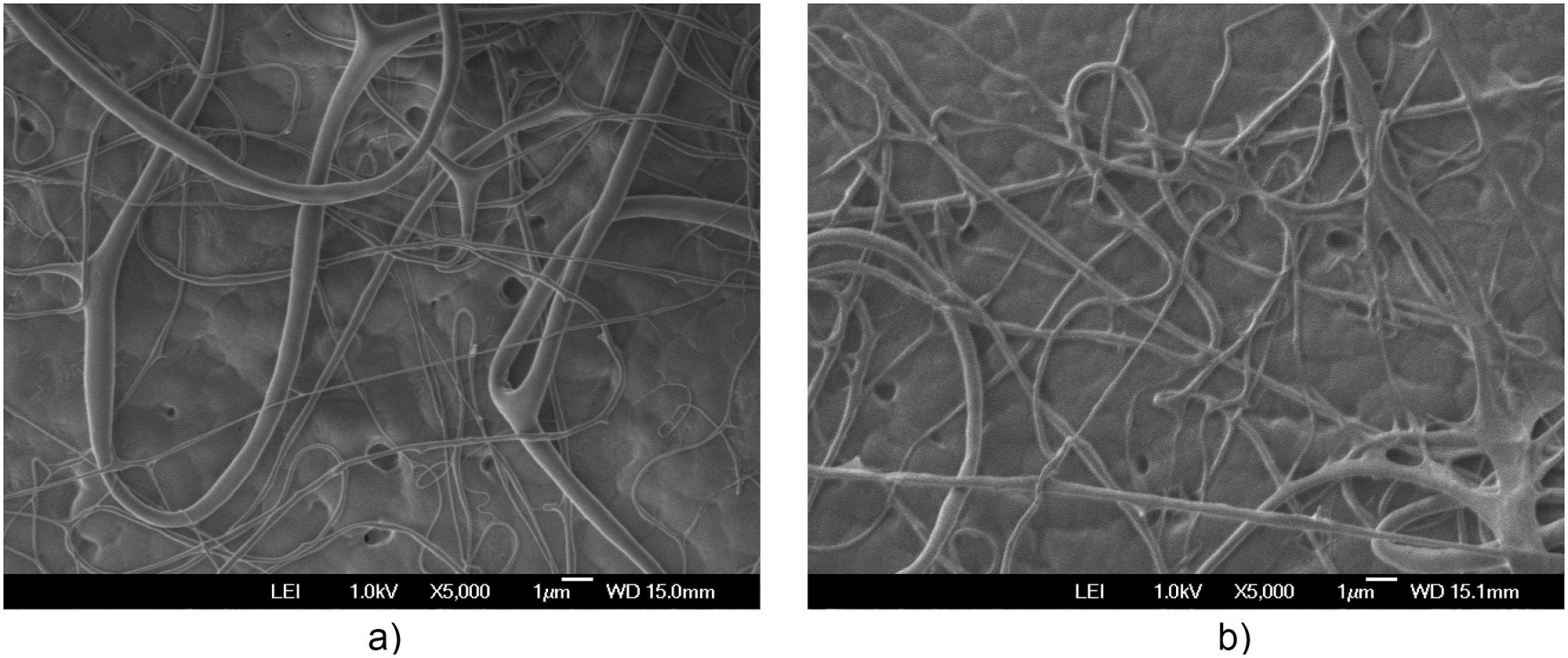
PVDF SEM x5000, a) central zone with electric field b) end zone without applying the electric field.

**Fig 15:**
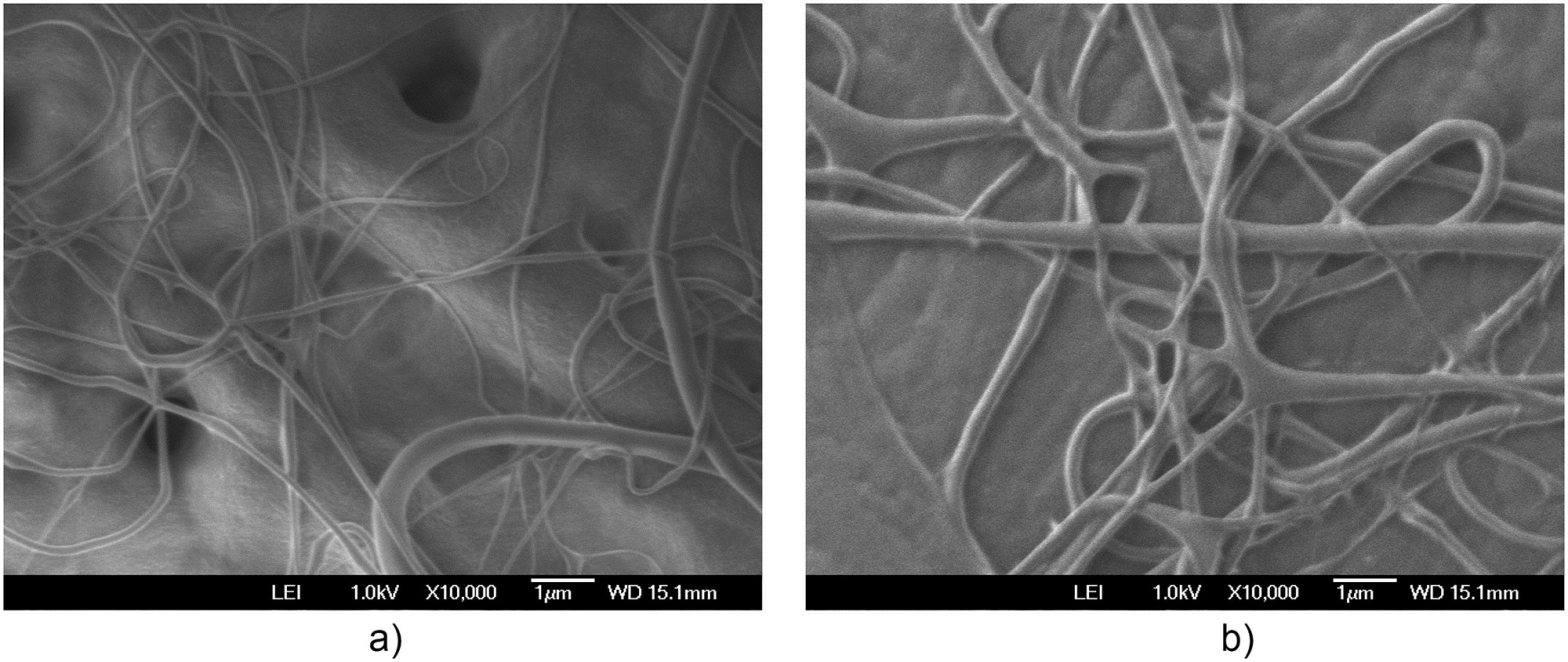
PVDF SEM x10000, a) central zone with electric field b) end zone without applying the electric field.

The additional electric field acting on the deflector plates is of low amplitude, -10 Vpp at a frequency of 1 to 10 Hz, compared to the voltage of 20,000 V in the positively polarized fiber jet between the Taylor cone anode (straight segment) and the collector-cathode, used in electrospinning. This additional electric field does affect the nanofiber diameter and orientation distribution. As shown in the amplified images obtained, the method described for preparing samples for PVC and PVDF membranes and a scanning electron microscope (SEM) JEOLJSM-6360LV were used. The Fig 12. PVC SEM x5000, Fig 13. PVC SEM x10000, Fig 14. PVDF SEM x5000, and Fig 15. PVDF SEM x10000 was used to determine the fiber diameter in relation to the percentage of nanofibers and the orientation distribution of the processed nanofibers. Also, these SEM figures exhibit in a) when the electric field was applied to the membrane obtained by electrospinning in the central zone through the deflector plates in the X or Y direction and in b) leaving it free without the application of the electric field to the plates in the extreme zone.

**Fig 16:**
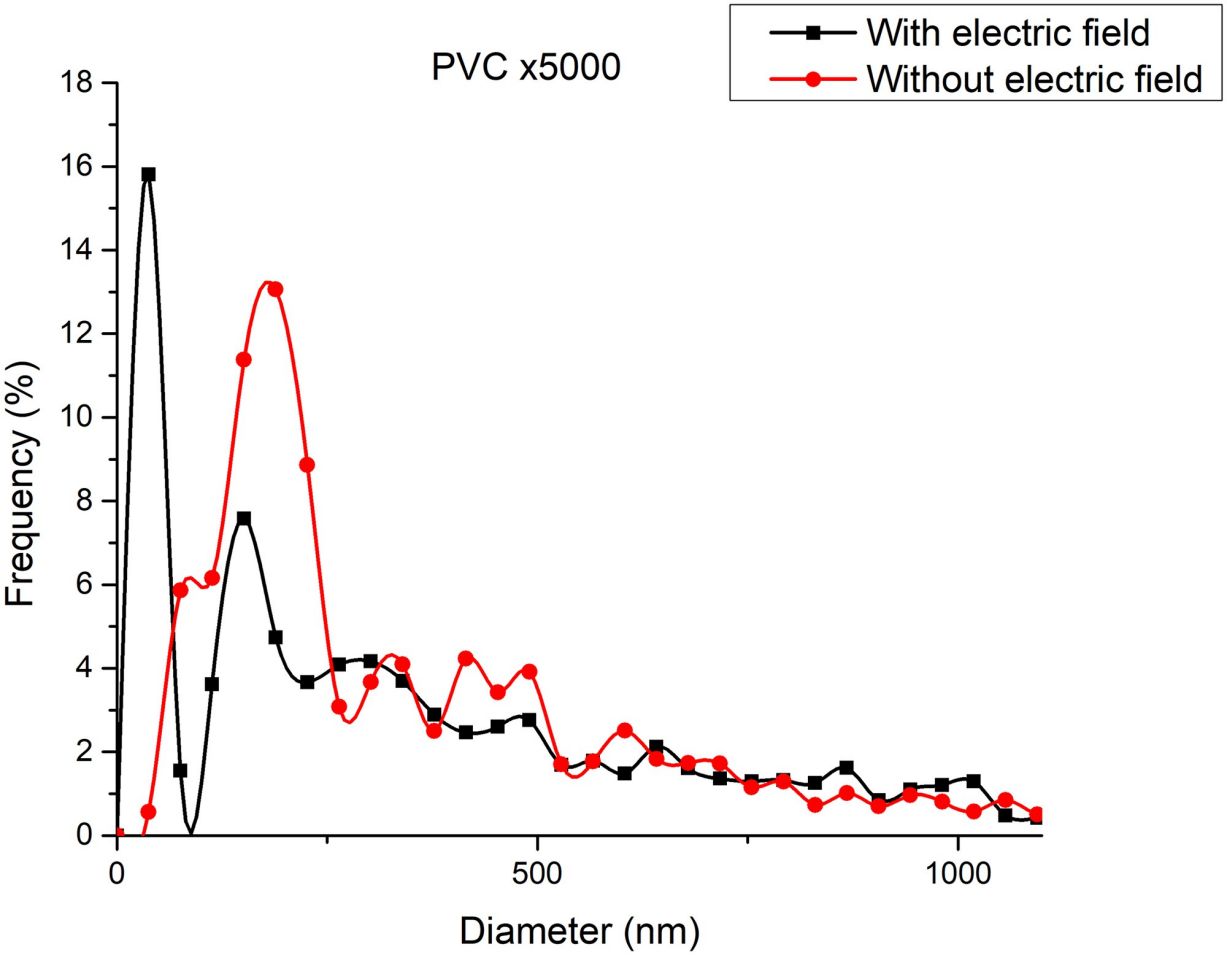
Frequency of fiber diameters in PVC SEM x5000.

Fig 16 shows the diameters of the fibers relative to the percentage of nanofibers in PVC SEM x5000, with and without applying an electric field.

**Fig 17:**
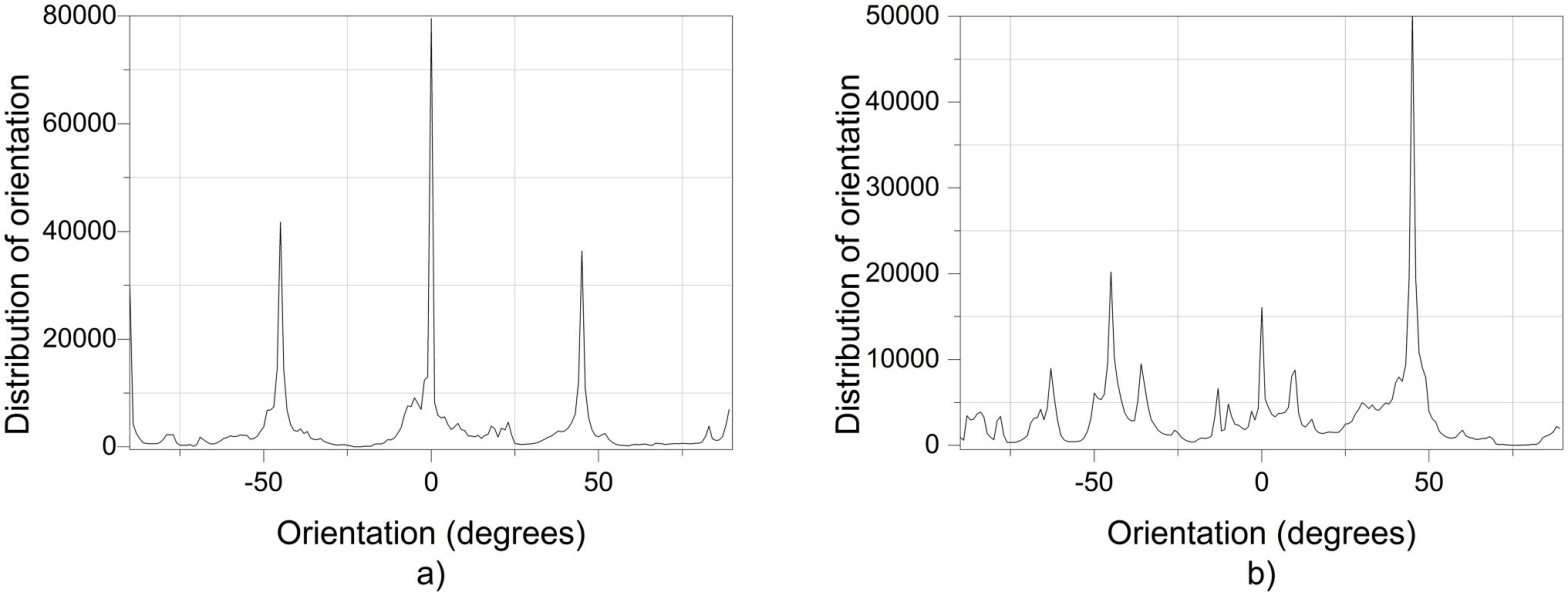
Orientation distribution of PVC nanofibers SEM x5000, a) With electric field, orientation of PVC SEM x5000, b)Without electric field, orientation of PVC SEM x5000.

Regarding the orientation of the nanofibers, the additional electric field had a significant impact. Fig 17 illustrates the orientation in degrees with respect to the center (0 degrees) of the PVC SEM x5000 image.

**Fig 18:**
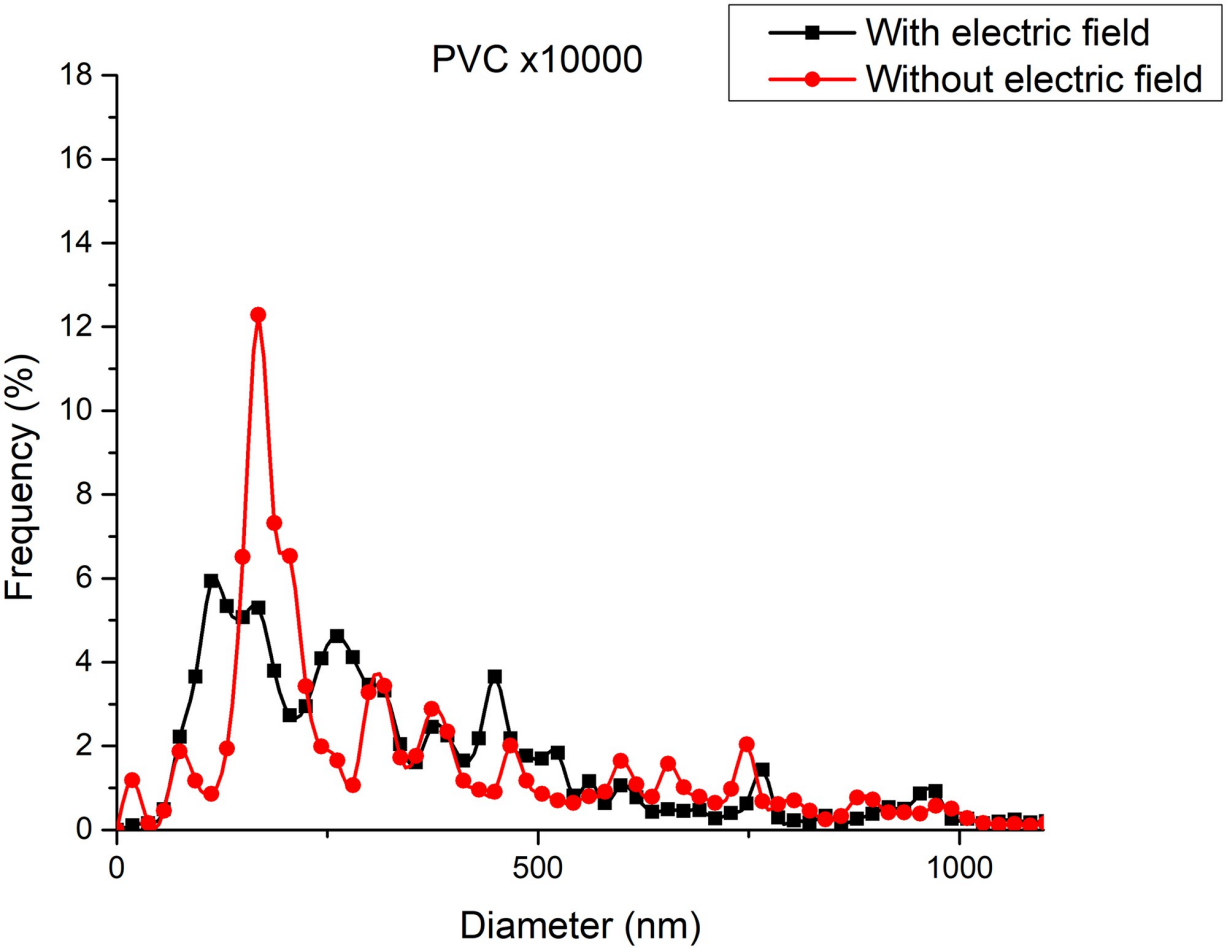
Frequency of fiber diameters in PVC SEM x10000.

Fig 18 shows the diameters of the fibers relative to the percentage of nanofibers in PVC SEM x10000, with and without applying an electric field.

**Fig 19:**
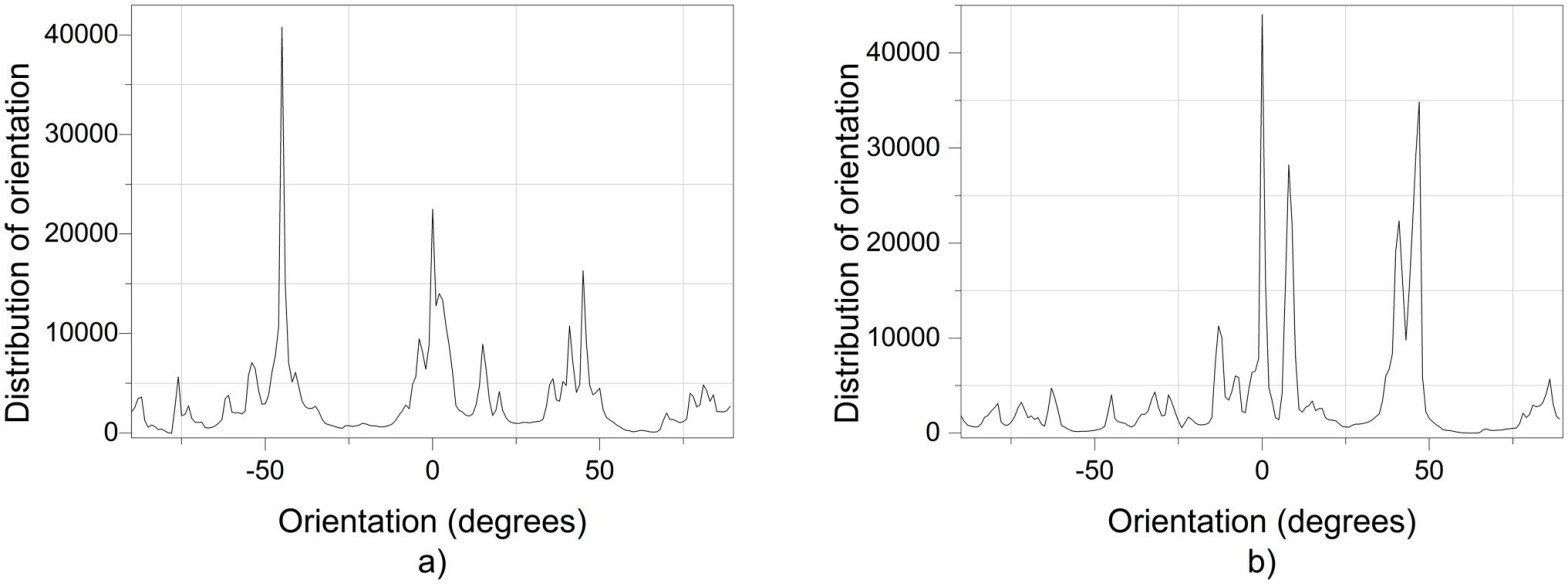
Orientation distribution of PVC nanofibers SEM x10000, a)With electric field, orientation of PVC SEM x10000, b)Without electric field, orientation of PVC SEM x10000.

Fig 19 illustrates the orientation in degrees with respect to the center (0 degrees) of the PVC SEM x10000 image.

**Fig 20:**
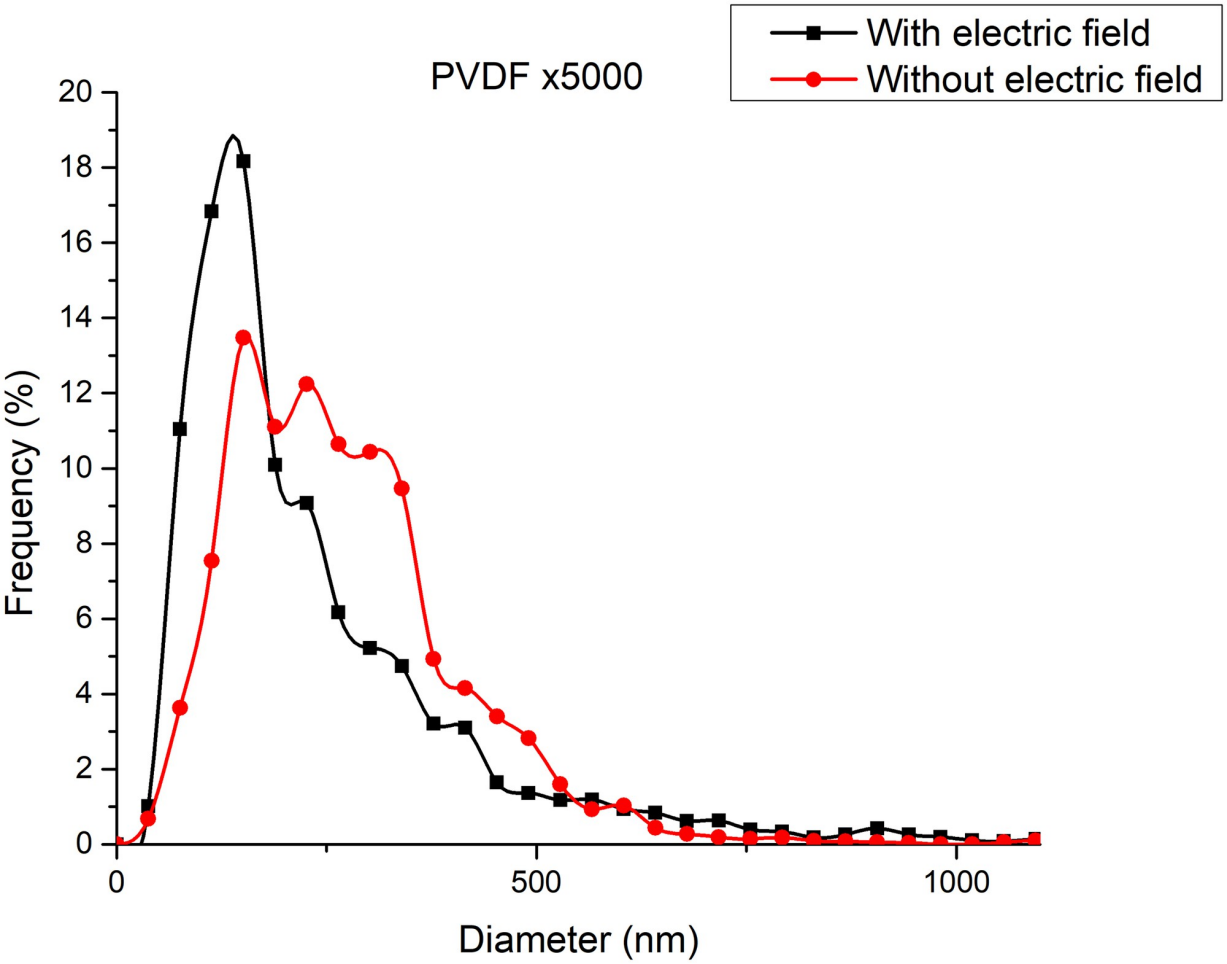
Frequency of fiber diameters in PVDF SEM x5000.

Fig 20 shows the diameters of the fibers relative to the percentage of nanofibers in PVDF SEM x5000, with and without applying an electric field.

**Fig 21:**
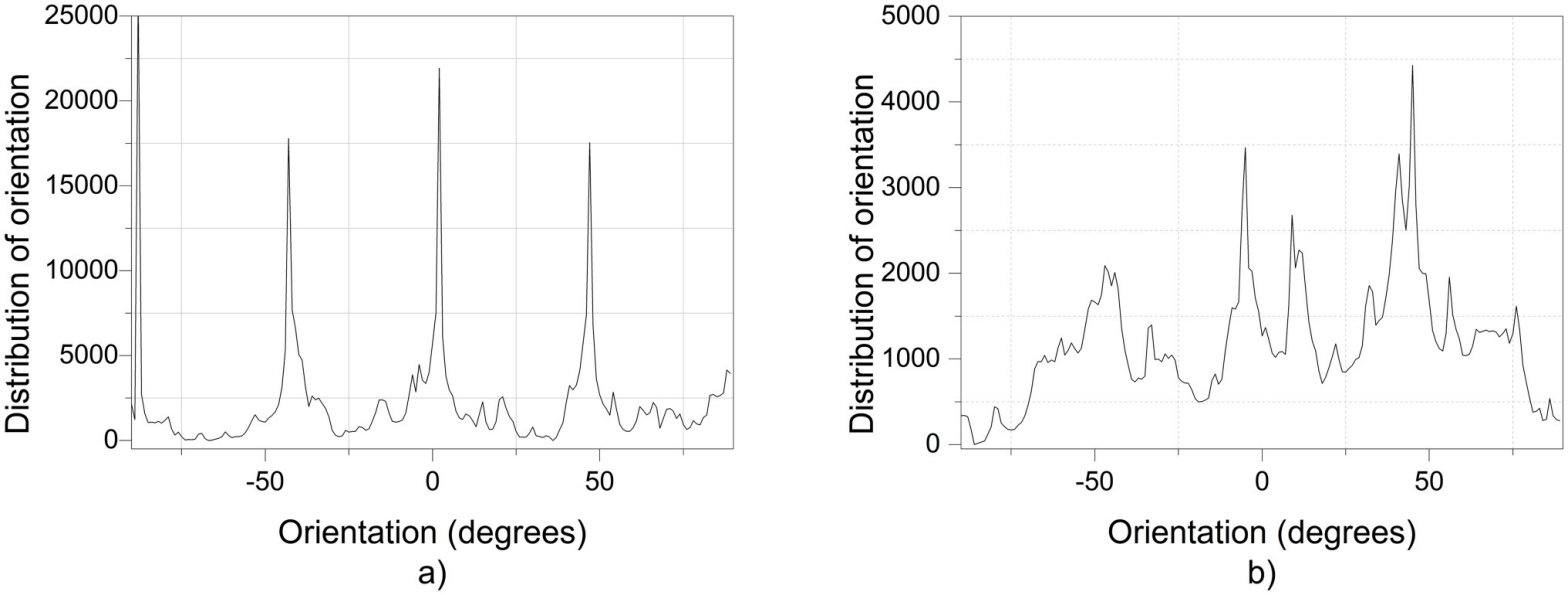
Orientation distribution of PVC nanofibers SEM x10000, a) With electric field, orientation of PVDF SEM x5000, b) Without electric field, orientation of PVDF SEM x5000.

Fig 21 illustrates the orientation in degrees with respect to the center (0 degrees) of the PVDF SEM x5000 image.

**Fig 22:**
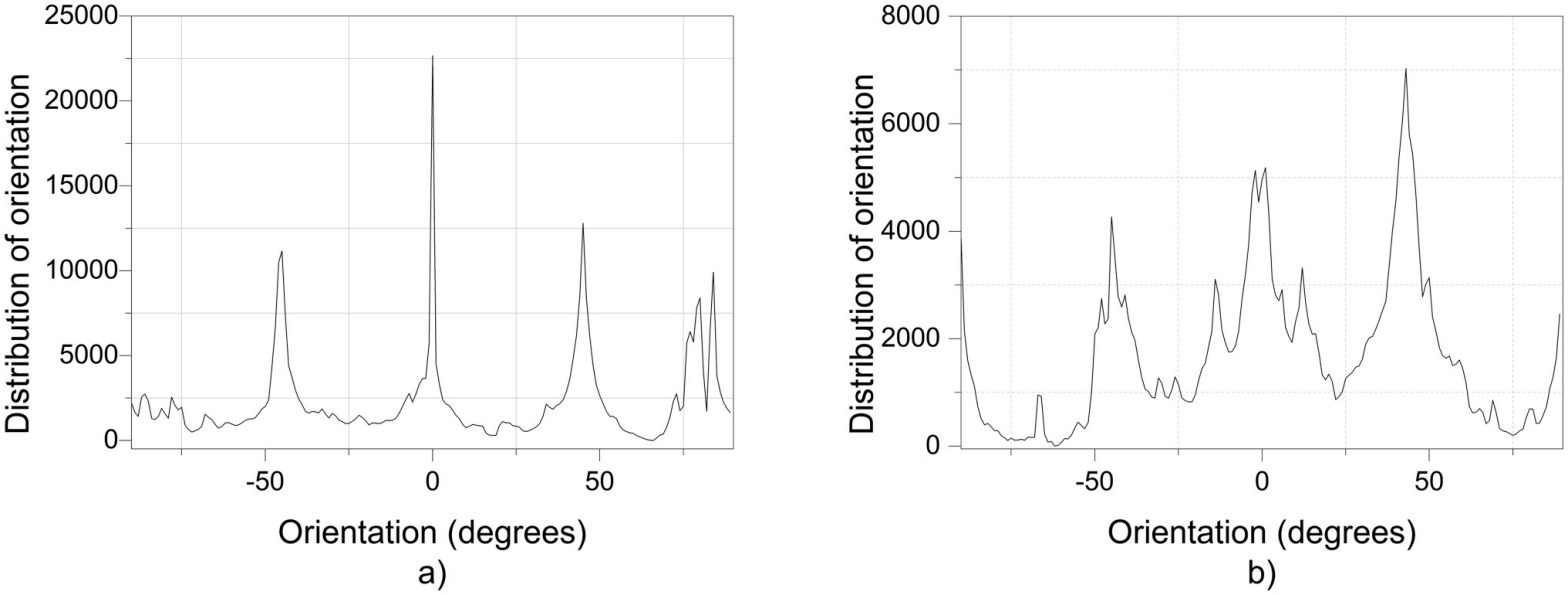
Orientation distribution of PVC nanofibers SEM x10000, a)With electric field, orientation of PVDF SEM x10000, b)Without electric field, orientation of PVDF SEM x10000.

Fig 22 illustrates the orientation in degrees with respect to the center (0 degrees) of the PVDF SEM x10000 image.

**Fig 23:**
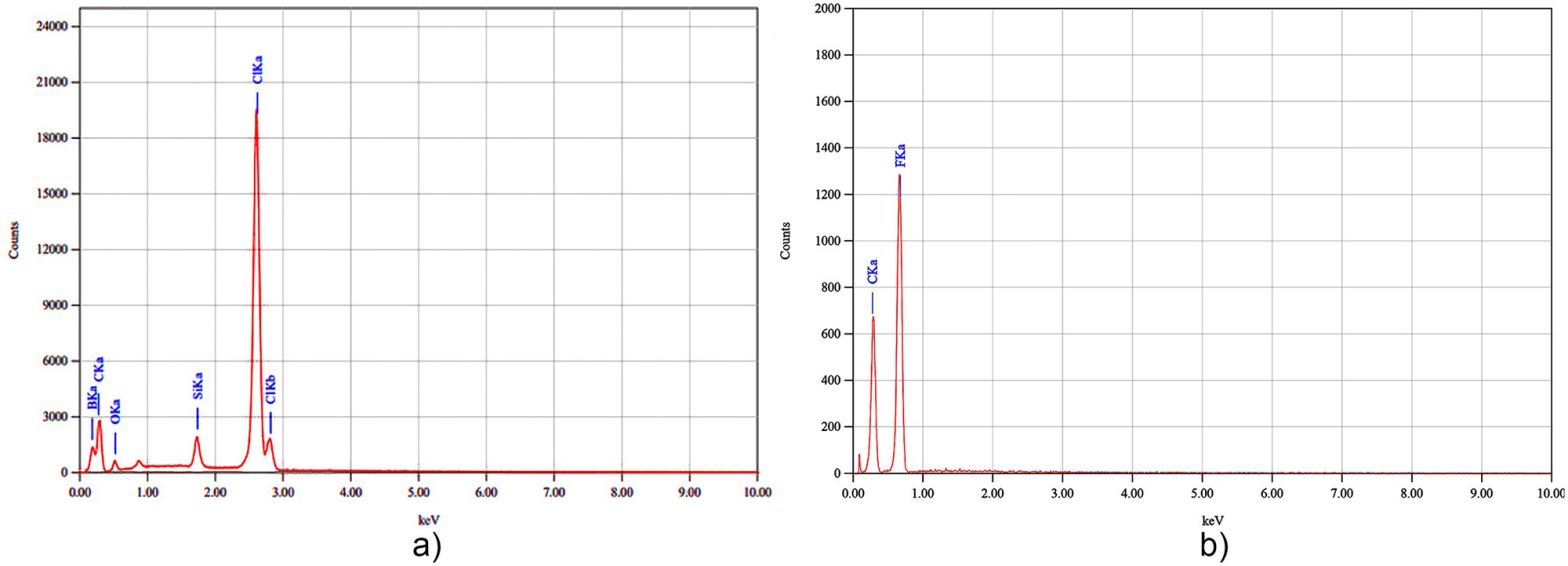
X-ray energy dispersive analyzer for: a) Membrane of PVC-based resin nanofibers and b) membrane of PVDF nanofibers.

The program described in [16] was used for processing nanofiber diameters and orientation distribution. Also illustrated in Fig 23, energy dispersive X-ray (EDS) analyzer [17, 18] for PVC-based resin and PVDF membranes from Figs 12 and 15 respectively.

## 4 Discussion

**Fig 24:**
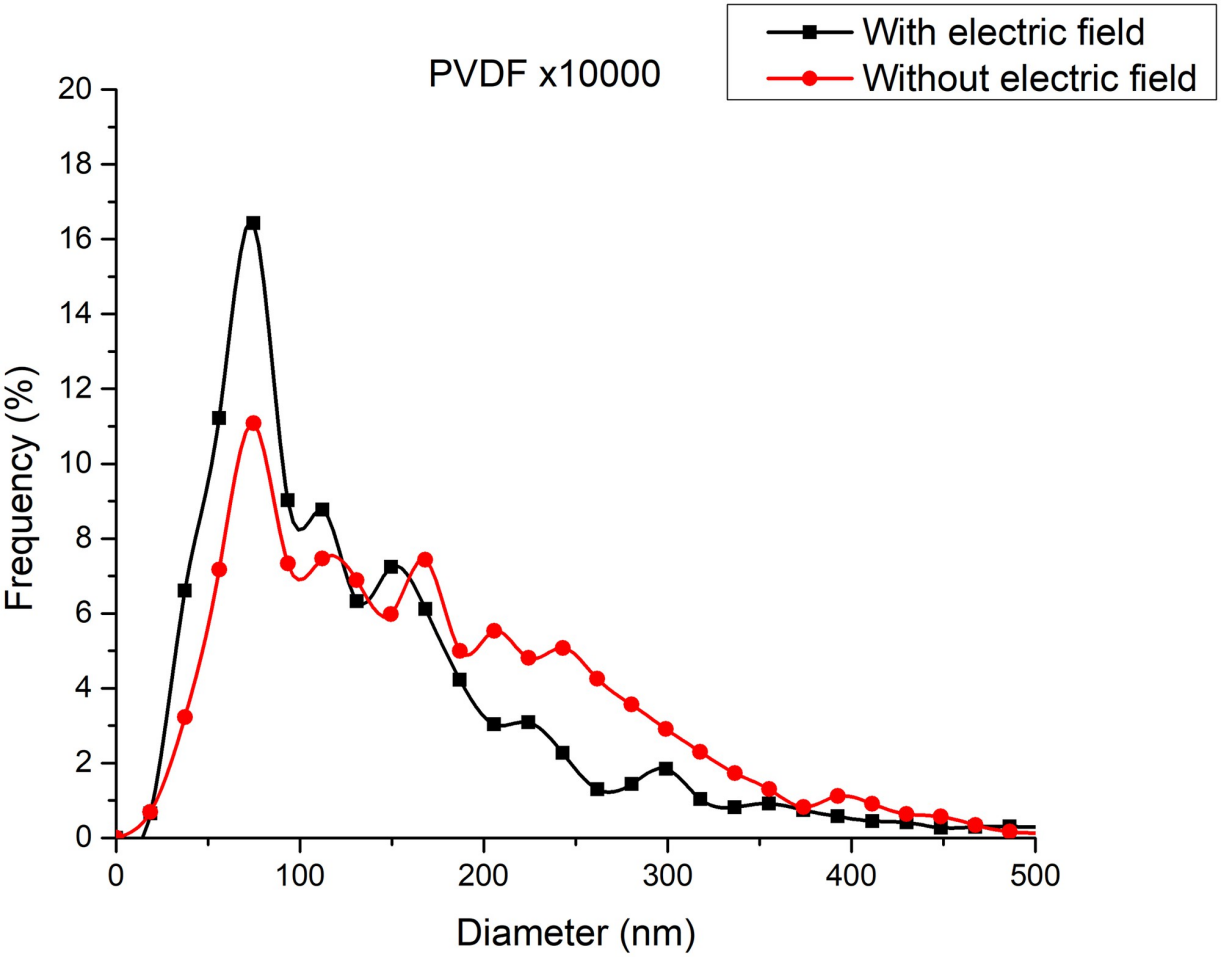
Frequency of fiber diameters in PVDF SEM x10000.

The development of polymeric membranes or “prints” of ultrathin branched PVDF and PVC fibers manufactured by modulated electrospinning and collimated by the induction of a variable negative electric field on the deflector plates acting on the jet of the polymer or positively charged substance. This methodology circumvents the potential for randomness in the trajectory within the membrane lattice. The method to create these non-randomly oriented membranes, which was proposed and experimentally tested, exhibited satisfactory results in modulating the nanofiber trajectory using an electrostatic deflection system as shown in Figs 5, 7 and 10, which acts on the fiber or substance of the positively charged jet which is presented at the beginning of the Taylor cone (Fig 4b). This electrostatic modulation system consists of a pair of deflecting metal plates for X and Y directions excited with a variable electric field with amplitude from -10 Vpp to 1 Hz. An 18 kV DC amplitude was used as the high voltage source of the electrospinning for 4 minutes. The thickness (Fig 11) or orientation Z was obtained by the superposition of membrane layers controlled by the electrospinning operation time. Also, the SEM images obtained by XYZ electrospinning of the PVC and PVDF membranes were used to determine the fiber diameter in relation to the percentage (Figs 16, 18, 20 and 24) and orientation distribution (Figs 17, 19, 21 and 22) of the processed nanofibers, with and without applying the electric field on the deflector plates. It was clearly demonstrated that the application of the electric field on the X-Y deflector plates did modify the diameter of the nanofibers, obtaining a fundamental range of the diameter of the nanofibers up to 375 nm, that is, Fig 16, up to 310 nm, Fig 18, up to 310 nm, Fig 20, up to 375 nm and Fig 24, up to 200 nm. Regarding the orientation of the nanofibers, the additional electric field had a significant impact, Figs 17a, 19a, 21a and 22a, illustrate the greatest orientation in degrees with respect to the center (0 degrees) at -45 to +45 degrees.

This equipment developed, XYZ electrospinning, opened the possibility for the first time to control at will the trajectory of the polymeric nanofiber bundles in the X-Y-Z directions, emulating a 3D printer. Thus, achieving any “printed” shapes or patterns of nanofiber bundles, with the potential for application in a wide range of industrial contexts [19–22], in the biomedical field [4, 6, 23, 24] as well as in medicine creating scaffolds or patches that supply the drug as well as in artificial skin with specific membrane sieves. The same can be said for the formation of arrangement of biosensors [7] in the form of a chessboard or emulating LF with different shapes and even in several layers of electrospinning [14]. Likewise, it can be used in the conformation of bounded arrays in specific trajectories with smart polymers such as piezopolymers, conductive polymers, or composites formed by specific polymers/oxides, such as ferroelectric, metallic as well as ferromagnetic polymer [25]. As for the formation of LF, which are complex and harmonic patterns, it is generally associated with the superposition of two simple harmonic motions in perpendicular directions [15], i.e., when sinusoidal signals are applied to both X and Y deflection plates. To obtain the circular LF, the phase difference between the two sinusoidal signals applied to the XY deflector plates was 90° or 270°. The potential of influencing the orientation of specific patterns of the polymer or substance jet by obtaining patterns or trends like LF deposited on the collector-cathode plate of the XYZ electrospinning (Fig 10) was explored.

## 5 Conclusions

The developed equipment, XYZ electrospinning, orients the trajectory and collimates the electrified fluid jet of polymer nanofibers by induced electric fields. It justifies the main conclusion: that by its characteristics and results obtained, it operates similarly to a 3D printer. With the control of a jet of the substance to be printed on the cathode with great versatility in the management of the position of the deflector plates XY, in the time of the deposit or thickness Z (Fig 11), in the value of the high voltage, of the distance between positively charged Taylor Cone and the cathode. Importantly, it is possible to emulate the production of electrospun membranes such as LF [14]. Furthermore, this innovative development creates a large area of high impact in biomedical engineering [5, 7, 23, 24] and industry [19, 20, 26], especially when used with jets of smart polymeric materials or nanofibers which respond to pressure, thermal, magnetic, light, chemical, flow, gas sensors, scaffolds, and permeable membranes, [5, 8, 21, 25]. Moreover, this development has opened up a myriad of uses when making electrospinning that emulate and follow trends with LF. Also, it has been essential to demonstrate and quantify in the SEM images obtained by XYZ electrospinning of the PVC and PVDF membranes the diameter of the fiber about the percentage and distribution of the orientation of the processed nanofibers, with and without applying the electric field in the XY deflector plates, which did modify the diameter and orientation distribution of the nanofibers.

Finally, a future direction and potential applications of XYZ electrospinning are proposed for the deflector plate electrodes by automating them through the control of linear actuators that operate during the electrospinning process, thereby controlling the deposition width. Another aspect would be to place a series of deflector plates, segment the electrodes, and excite the X-Y segments via a computer control program. Additionally, the high voltage distance between the Taylor cone and the cathode plate could be programmed to select fiber diameter. Therefore, the future direction would be to utilize different compounds for electrospinning, thereby creating combined membranes made from various materials with different X-Y positions and Z thicknesses. The significant impact of using XYZ electrospinning will be its versatility in making membrane deposits that can be managed or controlled by automated and programmed means, such as a 3D textile machine to produce different prints combined with different solutions.
